# Biosynthesis, Molecular Regulation, and Application of Bacilysin Produced by *Bacillus* Species

**DOI:** 10.3390/metabo12050397

**Published:** 2022-04-27

**Authors:** Tarequl Islam, Muhammad Fazle Rabbee, Jinhee Choi, Kwang-Hyun Baek

**Affiliations:** Department of Biotechnology, Yeungnam University, Gyeongsan 38541, Korea; tarequembg@gmail.com (T.I.); rabbi.biotech@gmail.com (M.F.R.); woolde@naver.com (J.C.)

**Keywords:** bacilysin, *Bacillus* species, biosynthesis, transcription regulator, glucosamine 6-phosphate synthase, quorum sensing

## Abstract

Microbes produce a diverse range of secondary metabolites in response to various environmental factors and interspecies competition. This enables them to become superior in a particular environment. Bacilysin, a dipeptide antibiotic produced by *Bacillus* species, is active against a broad range of microorganisms. Because of its simple structure and excellent mode of action, i.e., through the inhibition of glucosamine 6-phosphate synthase, it has drawn the attention of researchers. In addition, it acts as a pleiotropic signaling molecule that affects different cellular activities. However, all *Bacillus* species are not capable of producing bacilysin. The biosynthesis of bacilysin by *Bacillus* species is not uniform throughout the population; specificity and heterogeneity at both the strain and species levels has been observed. This review discusses how bacilysin is biosynthesized by *Bacillus* species, the regulators of its biosynthesis, its importance in the host, and the abiotic factors affecting bacilysin production.

## 1. Introduction

Antimicrobial peptides (AMPs) are short amino acid sequences produced by both unicellular and multicellular organisms to protect a host from pathogenic microbes, such as bacteria, viruses, fungi, and parasites [1]. Numerous AMPs have been identified that belong to two major classes, namely ribosomal AMPs, produced by all forms of life, and non-ribosomal AMPs, produced by bacteria, cyanobacteria and fungi [2,3]. Although all AMPs are produced by their hosts as a means of self-defense, they have significant clinical importance. For example, as resistance to traditional antibiotics is rapidly increasing, AMPs can be effective alternatives to traditional drugs [4]. The most beneficial features of these peptides over traditional antibiotics are that they act through multiple mechanisms of action, have a broad range of activity against microorganisms, and are less susceptible to resistance [4,5]. Positively charged AMPs typically interact with the negatively charged lipid head groups of the cytoplasmic membrane of the lipid bilayer, leading to the displacement of lipids [5,6]. Once internalized into the cytoplasm, these translocated AMPs can alter the cytoplasmic membrane resulting in the influx of water into the cell, loss of transmembrane potential and, finally, killing the bacteria [3,7].

*Bacillus* species are well known for producing a variety of AMPs, such as bacilysin, bacitracin, surfactin, fengycin, amylolysin, plantazolicin, bacillomycin D, lactosporin, and thuricin [8]. AMPs are associated with sporulation, germination, and several other cellular functions. For example, the peptide antibiotic tyrothricin inhibits RNA synthesis and RNA polymerase in *Bacillus brevis*. Thus, this peptide may be involved in gene regulation during sporulation [9]. A slightly different result was reported in the case of the peptide antibiotic gramicidin S, as it was observed that gramicidin S did not inhibit transcription during growth and sporulation, but it inhibited transcription during germination and outgrowth [10]. Sporulation was induced by supplementary peptide antibiotics in *B. brevis* when the cells were grown under low-nitrogen concentration in the culture medium [11]. In contrast to other AMPs, bacilysin acts as a signaling molecule either directly or indirectly and affects various cellular functions, as well as the spore quality [12]. A bacilysin-negative isolate of *B. subtilis* PY79 was obtained by *N*-Methyl-*N*′-nitro-*N*–nitrosoguanidine mutagenesis, which showed higher sensitivity to heat, chemicals, and lysozymes, as well as lower dipicolinic acid content than the wild-type strain [13,14]. The bacilysin influences quality of spores produced by *B*. *subtilis* PY79 since this effect was observed upon supplementation of bacterial culture with this compound before reaching the mid-log phase of growth [15].

Bacilysin is a dipeptide antibiotic compound with the molecular formula C_12_H_18_N_2_O_5_ and a molecular mass of 270.28 g/mol [16]. It is produced by aerobic spore-forming bacteria belonging to the genus *Bacillus*, and it causes cell lysis in bacteria and fungi [16]. This compound is mainly joined with an L-alanine residue at the *N*-terminus and L-anticapsin (a non-proteinogenic amino acid) at the C-terminus. The antimicrobial activity of bacilysin mainly due to the presence of L-anticapsin at the C-terminus [17]. Bacilysin was first discovered in *B. subtilis* in 1946 and was originally named bacillin [18]. Bacillin was highly active against Gram-positive and Gram-negative bacteria, such as *Staphylococcus aureus* and *Escherichia coli* [18]. In 1949, highly heat-stable bacilysin was reported from *B. subtilis* A14; however, it was not extractable using organic solvents [19]. In 1965, bacilysin was isolated from the culture filtrate of *B. subtilis* A14 at a very low yield [20]. When bacilysin was subjected to acid hydrolysis, it yielded L-alanine and L-amino acids [21]. In 1973, an antibiotic named tetaine synthesized by *B*. *pumilus* B-180 was shown to exhibit chemical and physical similarities to bacilysin [22]. In 1975, it was revealed that bacillin, bacilysin, and tetaine are identical compounds [23]. Bacilysin isolated from *B. subtilis* A14 was described as a heat-tolerant antibacterial compound that is stable between pH 1.4 and 12.0 for four hours at 20 °C and is insoluble in organic solvents [19].

Bacilysin is an antimicrobial compound that has attracted the attention of researchers owing to its simple structure and high antimicrobial activity against a broad range of microorganisms, including bacteria, fungi, and algae [16,17]. The *bac* operon (also referred to as *bacABCDEywf*G), which is responsible for bacilysin production, has been examined in molecular studies to understand bacilysin biosynthesis and molecular regulation in *B*. *subtilis* [24,25]. Another study observed the presence of antimicrobial activity using the same experimental procedure in the culture supernatant of *B. subtilis* A14 and *B. pumilus* B-180. The researchers concluded that bacilysin is produced by both strains at the beginning of the death phase [26,27]. 

This review aims to explain the mode of action of bacilysin, its role in the producer organisms, its biosynthesis by the *bac* operon, its molecular regulation, and the surrounding environment that influences bacilysin biosynthesis.

## 2. Mode of Action of Bacilysin and Inhibitory Effect on Pathogenic Microbes

**Figure 1:**
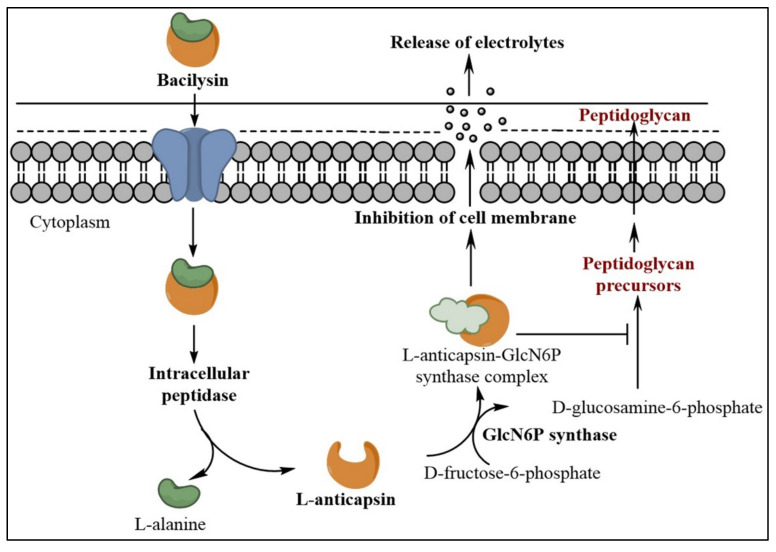
Mechanism of action of bacilysin during the inhibition of microbial cell wall biosynthesis.

Studies on the antimicrobial activity of bacilysin revealed that it inhibits glucosamine 6-phosphate (GlcN6P) synthase, which helps in the synthesis of GlcN6P from fructose-6-phosphate and glutamine, which is an essential component of the peptidoglycan of the bacterial cell wall [28]. As a result, bacilysin impaired formation of microbial cell wall [16]. Bacilysin itself has no antimicrobial activity. To be enzymatically active, bacilysin needs to be hydrolyzed by an intracellular peptidase that releases anticapsin, which inhibits GlcN6P synthase, an enzyme required for glucosamine or *N*-acetylglucosamine synthesis [29]. Intake of bacilysin into the cell and its hydrolysis into L-anticapsin and L-alanine are shown in Figure 1. Intracellular anticapsin blocks GlcN6P synthase and, hence, bacterial peptidoglycan or fungal mannoprotein synthesis is blocked, leading to cell protoplasting or cell lysis [29].

The inhibition of the enzyme GlcN6P synthase, which was heterologously expressed in *E. coli*, was studied using bacilysin. Bacilysin can be hydrolyzed by an intracellular peptidase of *C. albicans* and anticapsin is released into the cell [17]. Molecular simulation was used to verify the mechanism of action of bacilysin, which indicated that bacilysin is more easily transported into the cell than anticapsin. In addition, anticapsin forms a C–S bond with Cys1 of GlcN6P synthase, which is not formed with bacilysin [17]. Many studies have determined that GlcN6P synthase is an antifungal drug target [30]. Therefore, detailed information regarding the interaction between anticapsin and GlcN6P synthase will assist in the development of novel antifungal drugs [17]. 

Bacilysin synthesized by *B. amyloliquefaciens* FZB42 exhibited strong antibacterial activity against the cyanobacterium *Microcystis aeruginosa* (the causative agent of harmful algal blooms in lakes and rivers), with a killing efficiency of 98.78% [31]. Biosynthesis of this cyanobactericidal compound (i.e., bacilysin) was linked to the *aro* gene cluster, and the *sfp*-mutant strain CH03, unable to synthesize lipopeptides (LPs) or polyketides (PKs), was able to inhibit the growth of *M. aeruginosa*. This indicated the production of another antibacterial compound by *B. amyloliquefaciens* FZB42, which inhibited the growth of *M. aeruginosa*. Further studies showed that bacilysin produced by *B. amyloliquefaciens* FZB42 caused cell lysis and changed the membranes of many cell organelles of *M. aeruginosa*. Bacilysin has also been associated with the inhibition of harmful algae such as *Aphanizomenon flos-aquae*, *Nostoc* sp., and *Anabaena* sp. [31]. Bacilysin is easily transported to the target cells. Several dipeptides and tri-peptides in *S. aureus* are associated with bacilysin-uptake, whereas *E. coli* has both di- and oligopeptide transport systems [32]. Several dipeptides and tri-peptides in *S. aureus* NCTC 6571 compete to receive bacilysin [33].

*B. amyloliquefaciens* GSB272 transformed with plasmid pSB767 containing *bacABCDE* produced over ten times more bacilysin than the non-recombinant laboratory strain *B. subtilis* 168 [34]. These data demonstrate that *B. amyloliquefaciens* has a unique genetic backup for the production of bacilysin. This may be related to the presence of genes that positively regulate bacilysin biosynthesis [35,36,37]. Genomic analysis of *B. amyloliquefaciens* FZB42, also known as *B. velezensis* [38], showed the presence of gene clusters responsible for antibacterial PKs, such as difficidin and bacillaene, which act proficiently against *Erwinia amylovora*, a causative agent of fire blight disease [39,40]. A mutant strain unable to produce difficidin was able to remarkably suppress *E. amylovora* growth [39]. Moreover, 4′-phosphopantetheinyl transferase encoded by *sfp* plays a role in the production of lipopeptides (e.g., LPs and PKs); although mutants of this gene are unable to synthesize non-ribosomal LPs and PKs, the growth of *E. amylovora* is still suppressed. These results suggest that *B. amyloliquefaciens* has an antagonistic effect in suppressing *E. amylovora* growth [39]. A double mutant unable to produce PKs and bacilysin was not capable of suppressing *E. amylovora* growth, indicating that the additional inhibitory effect is due to the synthesis of bacilysin, and bacilysin biosynthesis is not dependent on *sfp* [39]. Bacilysin is synthesized via an SFP-independent non-ribosomal pathway. Bacilysin synthesized by *B. velezensis* FZB42 efficiently antagonizes *Phytophthora sojae*, which causes soybean root- rot disease. FZB42 mutants deficient in lipopeptides (bacillomycin D and fengycin) and PKs (bacillaene, difficidin, and macrolactin) did not impair antagonism against *P. sojae*. However, mutants deficient in bacilysin gene clusters completely lost their antagonistic effect against the pathogen, indicating the antifungal activity of this dipeptide antibiotic against the pathogen. Electron microscopy showed that bacilysin damaged the hyphal structures and loosened the cellular contents [41]. In a similar study, bacilysin synthesized by *B. velezensis* exhibited biocontrol potential against rice blight and leaf streak pathogens (*Xanthomonas oryzae* pv. *oryzae* and *X. oryzae* pv. *oryzicola*) [42].

The amount of bacilysin production depends on the nutritional composition of the growth medium and the growth conditions. Its GlcN6P synthase inhibitory activity decreases significantly in crude extracts of *S. aureus* when ethylenediaminetetraacetic acid (EDTA) inhibits the hydrolysis of bacilysin [29]. Moreover, bacilysin-resistant *S. aureus* strains have evolved altered cell surface receptors. Bacilysin-sensitive *S. aureus* strains utilize L-alanine produced by the hydrolysis of bacilysin within the cell, whereas resistant strains do not utilize it [29]. Bacilysin biosynthesis by *B. subtilis* 168 in synthetic medium containing sucrose and glutamate was also inhibited by certain growth conditions, such as growth supplements and temperatures above 30 °C [14,43]. The addition of usable carbohydrates to agar medium increased the production of bacilysin, whereas a reduced amount of carbohydrates decreased the rate of bacilysin synthesis in agar medium [18]. Antibacterial activity of *B. subtilis* against *E. coli* and *S. aureus* in yeast extract glucose agar medium was higher than that of yeast extract agar medium [18]. Moreover, the addition of asparagine to glucose agar medium dramatically increased the anti-*E. coli* activity of *B. subtilis* [18].

## 3. Strain Specificity of *Bacillus* Species in Bacilysin Production

The mechanism of bacilysin biosynthesis differs among various strains of *Bacillus* species. In addition to *B. subtilis* A14, many *Bacillus* species have been reported to synthesize bacilysin [34]. In silico genome analysis of several *Bacillus* species such as *B. amyloliquefaciens*, *B. velezensis*, *B. licheniformis*, *B. pumilus*, and *B. subtilis* revealed that the bacilysin gene cluster is present in all species, except *B. licheniformis* [44]. In a similar study, whole-genome sequencing data revealed that bacilysin gene clusters were common in several *Bacillus* species, including *B. velezensis* HNA3, *B. velezensis* FZB42, *B. amyloliquefaciens* DSM7, and *B. subtilis* 168 [45]. Bacilysin biosynthesis was not possible in *B. coagulans*, *B. licheniformis* ATCC 9789, and *B. megaterium* PV361 upon transformation with plasmid pSB660 containing *bacA*-*bacBCDEF*, which indicates that these genes are not solely responsible for bacilysin production [34]. In contrast, the transformation of vector pSB672 containing *bacABCD* genes or that of the vector pSB679 containing *bacAB* genes enabled bacilysin biosynthesis in *B. pumilus* ATCC12140 and *B. amyloliquefaciens* GSB272 [34]. 

*B. subtilis* showed wide variation in protein expression in the presence and absence of the *bac* operon. Both gel- and gel-free proteomics analysis were performed to observe differences in protein expression. Based on these findings, it was concluded that bacilysin acts as a pleiotropic signaling molecule that affects different cellular activities [12]. Similar results have been reported for the pleiotropic gene *scoC* in *B. pumilus* BA06 and *B. subtilis* genomes, which causes transcriptomic and phenotypical changes [46,47]. Mutant strains with disrupted *scoC* gene in *B. pumilus* BA06 increased total extracellular protease activity and reduced cell motility, as flagella formation was affected. Transcriptome analysis showed that more than a thousand genes were altered during multiple growth stages at the transcription level, including many protease genes, particularly the *aprE* gene [46]. In *B. subtilis*, ScoC was reported to regulate at least 560 genes [47]. Moreover, *B. pumilus* also downregulated the *aprN* gene encoding a neutral protease in the *scoC* mutant, indicating that ScoC plays a strain-specific role [46]. AbrB, DegU, ScoC, and SinR are also reported to be associated with the extracellular expression of AprE and NprE proteases in *B. subtilis* [48]. Production of bioactive compounds by *Bacillus* species differs at both the strain and species levels [49]. Presently, different regulators and environmental factors involved in bacilysin biosynthesis are summarized, although many other unknown peptide antibiotics are now being produced by different *Bacillus* species.

## 4. Biosynthesis of Bacilysin by the *bac* Operon

**Figure 2:**
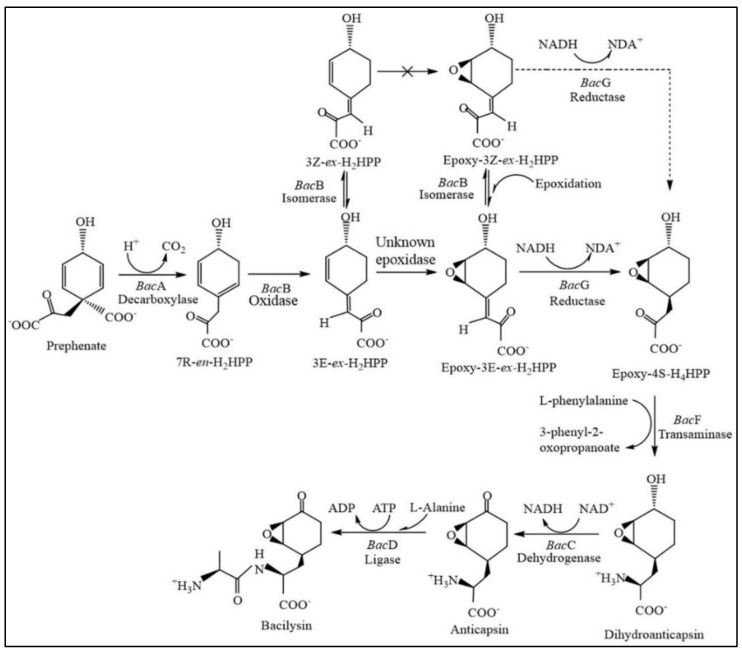
Bacilysin biosynthesis pathway according to Parker and Walsh [24].

Bacilysin biosynthesis is governed mainly by the *bac* operon, which plays a key role in the conversion of prephenate to bacilysin. Some regulatory genes also regulate the expression of the *bac* operon, which is discussed in the latter part of the article. This complex and unique genetic setup of *B. subtilis*, *B. amyloliquefaciens*, and *B. pumilus* enables them to produce bacilysin more efficiently than any other *Bacillus* strain. The bacilysin biosynthesis pathway starts from prephenate with the help of genes of *bac* operon (Figure 2).

The enzyme encoded by *bacA* decarboxylates prephenate without aromatization, converting the 1, 4-diene in prephenate to endocyclic 1, 3-diene in 3Z-ex-H_2_HPP. The *bacA* gene was cloned and expressed in *E. coli,* and the purified homogenized recombinant protein was incubated with a labeled substrate [50,51]. The results showed that the enzyme was stereo-selective and created only the (R)-isomer of the C7-hydroxyl group. The *bacB* gene was cloned and expressed in *E. coli,* and the purified homogenized recombinant protein was incubated with a labeled substrate [52]. The gene encodes an isomerase that acts on 3Z-ex-H_2_HPP. Moreover, it can convert the (E) isomer into the (Z) isomer and vice versa [53]; although only the (E) isomer is used in the bacilysin biosynthesis pathway [24]. The encoded enzyme has oxidase activity and acts on 7R-en-H_2_HPP, converting it to 3E-ex-H_2_HPP or 2-oxo-3-(4-oxocyclohexxa-2,5-dienyl) propanoic acid, which is the precursor of L-anticapsin [54]. A mutant lacking *bacB* was unable to produce L-anticapsin or bacilysin, indicating that the end product of *bacB* leads to the production of L-anticapsin and bacilysin [24].

The *bacG* gene of *B. subtilis* is an integral component of the bacilysin biosynthesis gene cluster. The gene was cloned and expressed and the product of the gene was purified and characterized similarly to *bacA* or *bacB* [52]. A previous study showed that the introduction of an epoxy moiety is required for the enzymatic activity of *bacG*. Therefore, the substrate for *bacG* is epoxy-3E-H_2_HPP, which is converted to epoxy-4S-H_4_HPP by the end of this reaction [55,56]. 

Another essential component of the bacilysin biosynthetic gene cluster is *bacF*. BacF protein is a fold-type I pyridoxal 5-phosphate (PLP)-dependent stereospecific transaminase [57]. This enzyme uses l-phenylalanine to donate an amino group to oxidize the 2-keto group of 3- (4-hydroxyphenyl) pyruvate, producing l-tyrosine. The enzyme uses epoxy-4S-H_4_HPP as its substrate, converting it to l-dihydroanticapsin, which is the precursor of L-anticapsin [55,56].

*bacC* encodes a dehydrogenase or reductase that oxidizes the C4-hydroxyl of L-dihydroanticapsin, which takes place immediately after the cyclohexenol double bond epoxidation. Mutants with *bacC* deficiency were unable to synthesize L-anticapsin or bacilysin, suggesting that it is an essential element for bacilysin production. Computational analysis of BacC proved that it is a member of the NAD^+^-dependent oxidoreductase family [24]. 

The *bacD* gene of the *bac* operon encodes an amino acid ligase. It was previously investigated and determined to be an unorganized dipeptide ligase [24]. BacC (oxidase) and BacD (ligase) are the last enzymes in the biosynthesis of bacilysin. Dihydroanticapsin and dihydrobacilysin found in ΔbacC strain were converted to anticapsin and then bacilysin, respectively, upon addition of BacC and BacD, respectively. These findings suggest that the epoxide group in bacilysin is installed early in the biosynthetic process, while BacC oxidation of the C7-hydroxyl and subsequent BacD ligation of anticapsin to l-Ala are the two last steps of this process [24].

## 5. Regulatory Role of Signaling Molecules in Bacilysin Biosynthesis

**Figure 3:**
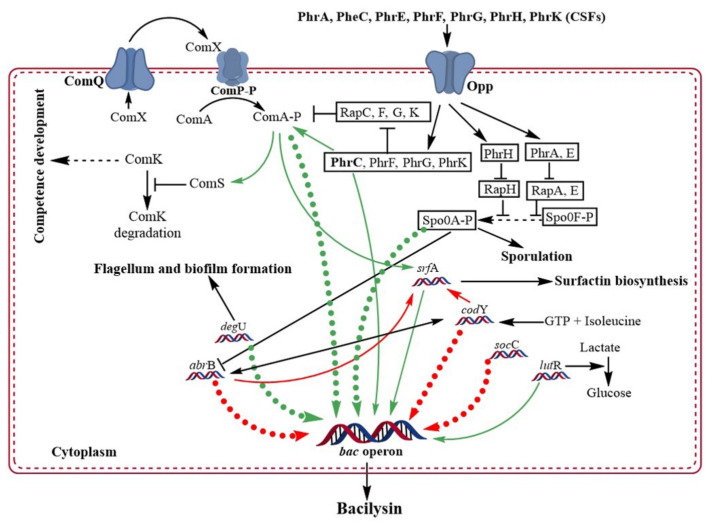
Transcription regulators that control bacilysin biosynthesis. Red bold dotted arrows indicate regulators that directly bind to the *bac* promoter and negatively regulate bacilysin biosynthesis, whereas green bold dotted arrows indicate regulators that regulate bacilysin biosynthesis positively by binding to the bac promoter. Other red and green arrows indicate regulators involved in indirect negative and positive regulation, respectively.

Bacteria produce extracellular signaling molecules at high cell densities that are involved in drastic changes in gene expression through a mechanism known as quorum sensing (QS). QS is the bacterial response or communication at high cell concentrations that allows them to control specific processes through gene regulation. QS governs antibiotic production, sporulation, and competence development in all *B. subtilis* strains via a pathway known as ComQXPA [55]. Two QS pathways organize molecular competence in *B. subtilis*. First, the Com signaling pathway is composed of ComP-ComA, which is a two-component regulatory system pathway triggered by the ComX pheromone. Second, the Phr–Rap signaling pathway, which is triggered by a small oligopeptide permease (Opp) [56]. ComX and competence-stimulating factor (CFS) are two extracellular signaling proteins in *B. subtilis*. At the beginning of this procedure, cell-derived pheromones ComX and CSF (also known as PhrC) accumulate outside the cells [56,58,59,60]. ComX is a 9–10 amino acid peptide that activates ComP (a membrane-attached receptor protein kinase of ComX) by phosphorylation, which further activates ComA (Figure 3) [58,61]. ComX, ComP, and ComA affect the same gene sets. The Com signaling pathway directly controls the expression of over twenty genes and indirectly controls the expression of over 150 genes, including competence-developing genes [56]. RapC is a member of the Rap protein family that encodes a 382 amino acid protein, aspartate phosphatase, which is a response regulator that controls ComA activity [62]. RapC is a negative regulator of ComA, which removes the phosphate group from ComA, making it inactive [58,63]. CSF is transported back into the cell by oligopeptide permease (Opp) [63,64]. CSF, a five amino acid extracellular signaling peptide, also activates ComA by inhibiting RapC activity [62]. Activated ComA acts as a multifunctional transcriptional activator and regulates QS in *B. subtilis* [65]. Bacilysin production is controlled by the complex regulatory mechanisms of ComX, PhrC, CSF, and ComP/ComA in *B. subtilis* through QS [35,37].

Opp is significantly involved in bacilysin production via the QS pathway and handles sporulation, competence development, and the initiation of surfactin production [35]. Opp impairment results in a bacilysin-negative phenotype [35]. The function of peptide pheromones (Phr peptides) verified the involvement of Opp in bacilysin biosynthesis. Phr peptides are extracellular signaling molecules that enter cells with the help of Opp [66]. To verify the role of Phr peptides (PhrA or PhrC) in bacilysin biosynthesis, *phrA*, *phrC*, and *comA* deletion mutants of *B. subtilis* PY79 were constructed, and the results showed that these genes depend on Opp for proper function [35]. An insertion mutation in *phrC* resulted in a bacilysin-negative phenotype in *B. subtilis* PY79 [35]. Out of the 50 transformed cells, 41 cells were bacilysin-negative and 9 were slightly bacilysin-positive. Insertion instability may cause the slight bacilysin positivity in these nine cells of *B. subtilis* PY79. In addition, an insertion mutation in *comA* also caused the same result, where 43 out of 50 transformed cells were phenotypically bacilysin-negative [35]. In contrast, an insertion mutation in *phrA* did not reveal any relationship with bacilysin production [35]. This result indicated that PhrC and ComA are involved in bacilysin production in an Opp-dependent manner. 

A group of Phr peptides (PhrA, PhrE, PhrC, PhrF, PhrG, and PhrK), induces sporulation and competence development [67,68]. The first two peptides are involved in sporulation, whereas the remaining peptides are involved in the development of competence. PhrH is another peptide in this group that is involved in both sporulation and competence development [56,69]. Genes encoding the Phr peptides are transcribed with the help of the Rap operon, in which the signaling pathway is coupled with ComX [66]. ComX controls ComA activity and directly influences the expression of the *bac* operon. The signaling pathway of Phr peptides is also coupled with ComX. Together with these data, it can be hypothesized that Phr peptides may be involved in the expression of the *bac* operon [35]. When the *bacA*-*lacZ* fusion was expressed in a mutant lacking *phrC*, *phrF*, and *phrK*, a huge variation in the expression of the *bac* operon was found [70]. Most significantly, deletion of *phrC* results in complete cessation of the *bac* operon [70]. In contrast, the expression of the *bac* operon is possible in some strains without ComX. Therefore, it can be concluded that ComX-mediated signaling is strain specific, whereas PhrC is species specific. The addition of PhrC could compensate for ComX-mediated signaling in deficient strains of *B. subtilis* [71].

The *srf*ABCD operon of *B. subtilis* encodes surfactin, a non-ribosomally synthesized LPs known to act against several pathogenic microbes, including *L. monocytogenes*, *Enterococcus faecalis*, *S. aureus*, *Pseudomonas aeruginosa*, *E. coli*, *Fusarium oxysporum*, *F. moniliforme*, *F. solani*, *Trichoderma atroviride*, and *T.* reesei [72,73,74]. SrfA has a direct effect on bacilysin biosynthesis in *B. subtilis* PY79 [37]. To verify this, *srfA* mutant isolates were investigated; they could express *bacA*-*lacZ*, but the expression of the *bac* operon was not observed [37]. AbrB is a transcription regulator of cells, which negatively regulates the transcription of many genes, including *srfA* [73,75], and has a direct impact on bacilysin biosynthesis by *B. subtilis. B. subtilis* that lacked *spo0H* and/or *spo0A* (repressor of the *abrB* gene) could not produce bacilysin, whereas blocking AbrB significantly increased bacilysin production in the mutant strain [37]. Spo0A directly interacts with the *bac* promoter and positively and indirectly regulates its expression and enhances the expression of the *bac* operon by suppressing the *abrB* gene. When Spo0A directly binds to the *bac* promoter, AbrB cannot bind to the promoter; thus Spo0A indirectly regulates the expression of the *bac* operon positively by inhibiting AbrB from binding to the *bac* operon [70]. *B. subtilis* strains that could not produce bacilysin were suppressed by an *abrB* mutation in *spo*0*A*-blocked mutants. All these reports suggest that gene transcription for bacilysin biosynthesis is negatively controlled by AbrB and is relieved by Spo0A [70]. 

Intracellular GTP levels are directly related to the *bac* operon. A decrease in GTP level results in improved expression of the *bac* operon [36]. In wild-type *B. subtilis*, the addition of decoyinine (an inhibitor of GMP synthetase) enhanced the expression of the *bac* operon, resulting in a 2.5 fold increase in bacilysin biosynthesis [36]. CodY is a global transcriptional regulator in low G+C containing Gram-positive bacteria that controls over 200 genes in *B. subtilis*, encoding peptide transporters, intracellular proteolytic enzymes, and amino acid degradative pathways, along with the stationary phase and virulence [76,77]. Interaction between GTP and isoleucine activates CodY, which enhances its affinity for its target sites [77]. CodY, a transcriptional regulator, controls intracellular GTP levels. Expression of the *bac* operon was increased in the mutant strains of *Bacillus* spp. lacking *codY* gene, suggesting that its product negatively regulates transcription of these genes [36]. Another study reported that AbrB and CodY do not directly repress the *bac* operon; however, both can bind to the promoter region of the *bac* operon. As a result, they act mutually to bind to the *bac* operon and do not interfere with each other’s activity [70].

ScoC (*hpr*) negatively regulates protease synthesis and sporulation in *B. subtilis* [78]. Genomic comparison of a large number of bacilysin-producing *B. subtilis* strains revealed that they all have *scoC* mutation. The expression of the *bac* operon was higher in mutants lacking *scoC*, and it has been identified that ScoC directly binds to the promoter of the *bac* operon and, with AbrB and CodY, negatively regulates the transcription of the *bac* operon [79]. However, CodY can minimize the regulatory activity of ScoC [48]. The above information reveals that the three transcriptional regulators (ScoC, AbrB, and CodY) can bind to the *bac* promoter and negatively regulate its transcription, while ComA and Spo0A positively regulate the transcription of the *bac* operon.

The expression of the *bac* operon in *B. amyloliquefaciens* FZB42 is also positively regulated by the *degU* gene, which encodes the transcriptional regulatory protein DegU. It is associated with various cellular functions and gene regulation in *B. subtilis*. Both phosphorylated and unphosphorylated forms of this protein are active and regulate different gene functions [80,81]. Similar to ScoC, DegU binds to the *bac*A promoter. It regulates *bac*G, an integral gene in bacilysin biosynthesis [82]. GntR, a large family of transcription factors found in *B. subtilis*, has four subfamilies categorized on the basis of their effector-binding domains. It has two additional regulators: LutR and YdhC. It has also been demonstrated that LutR (also known as YvfI) is essential for bacilysin biosynthesis [83]. A mutant strain of *B. subtilis* PY79 in which nucleotides 196–314 of the *lut*R gene was deleted resulted in a bacilysin-negative phenotype. In contrast, mutations in the *lacR* gene located downstream of the *lutR* gene did not affect bacilysin biosynthesis. These results indicate that LacR does not influence bacilysin biosynthesis, whereas LutR is involved in bacilysin biosynthesis [83]. All genes and gene products involved in bacilysin biosynthesis and regulation are listed in Table 1. 

## 6. Conclusions and Future Prospects

The information presented above demonstrates how bacilysin is synthesized in *Bacillus* species and how bacilysin biosynthesis is regulated at the molecular level. The review also reveals how bacilysin production and the expression of several extracellular proteases are controlled in *Bacillus* species by investigating studies on the involvement of various transcriptional regulators or pleiotropic signaling molecules. However, the control mechanism of bacilysin biosynthesis in *Bacillus* species and its function are still unclear. For example, it is well known that bacilysin inhibits GlcN6P synthase; however, which proteins are involved in its trafficking inside the cell, or which amino acids are involved in the interaction between GlcN6P synthase and anticapsin-GlcN6P complex formation is unknown. Moreover, bacilysin extraction from its producers in its original form is very challenging. Thus, more research is needed to ensure that bacilysin can be extracted efficiently without losing its biological function. In addition, *Bacillus* species that produce high amounts of bacilysin can be genetically manipulated to enhance bacilysin production. In the future, this approach might be a potential method for producing long-lasting biocontrol agents using *Bacillus* spp. for sustainable agriculture.

## Figures and Tables

**Table 1:** Genes involved in bacilysin biosynthesis and its regulation.

Genes	Gene Product Sizes (aa)	Gene Products	Functions of Gene Products	References
Genes directly involved in bacilysin biosynthesis				
*bac*A	204	Decarboxylase	Acts on prephenate	[24,50,51]
*bac*B	235	3E-ex-H_2_HPP isomerase	Synthesizes epoxy-3E-H_2_HPP	[50,51,52]
*bac*C	255	Dehydrogenase	Synthesizes L-anticapsin	[24,52]
*bac*D	472	Ligase	Ligases L-anticapsin and L-alanine	[24,34]
*bac*E	394	Bacilysin exporter	Provides host resistance to bacilysin and effluxes it from cell	[34]
*bac*F	399	Aminotransferase	Synthesizes L-dihydroanticapsin from L-phenylalanine	[24,51,79]
*bac*G	259	Reductase	Synthesizes epoxy-4S-H_4_HPP precursor of L-anticapsin	[24,51]
Genes positively regulate bacilysin biosynthesis				
*srf*A	3588	Surfactin synthase subunit 1	Regulates bacilysin biosynthesis positively.	[24,84]
*deg*U	229	Transcriptional regulatory protein DegU	Binds *bac*A operon and *bac*G genes	[82]
*com*X	55	Competence pheromone ComX	Activates *com*A which positively regulates *bac* operon (Quorum sensing).	[56]
*phr*C	40	Phosphatase	Controls *com*A activity by blocking RapC	[56,67]
*lutR*	219	HTH-type transcriptional regulator LutR	Controls lactate utilization, regulates *bac* operon positively	[80,83]
Genes negatively regulate bacilysin biosynthesis				
*soc*C	203	Deoxyfructose oxidoreductase	Negatively control the expression of *bacA* gene	[79]
*abr*B	96	Transition state regulatory protein AbrB	Binds to the bac operon and regulates bacilysin biosynthesis negatively. Acts mutually with CodY	[37,75]
*cod*Y	259	Transcriptional regulatory protein CodY	Binds to the bac operon and regulates bacilysin biosynthesis negatively. Acts mutually with abrB	[36,70]

Note: aa: amino acids.

## Data Availability

Not applicable.

## References

[1] Clark S., Jowitt T.A., Harris L.K., Knight C.G., Dobson C.B. (2021). The lexicon of antimicrobial peptides: A complete set of arginine and tryptophan sequences. Commun. Biol..

[2] Kleinkauf H., Von Döhren H. (2008). Peptide Antibiotics. Biotechnology.

[3] Li J., Koh J.J., Liu S., Lakshminarayanan R., Verma C.S., Beuerman R.W. (2017). Membrane active antimicrobial peptides: Translating mechanistic insights to design. Front. Neurosci..

[4] Benfield A.H., Henriques S.T. (2020). Mode-of-action of antimicrobial peptides: Membrane disruption vs. intracellular mechanisms. Front. Med. Technol..

[5] Palmer N., Maasch J.R.M.A., Torres M.D.T., De La Fuente-Nunez C. (2021). Molecular dynamics for antimicrobial peptide discovery. Infect. Immun..

[6] Fjell C.D., Hiss J.A., Hancock R.E.W., Schneider G. (2012). Designing antimicrobial peptides: Form follows function. Nat. Rev. DrugDiscov..

[7] Brogden K.A. (2005). Antimicrobial peptides: Pore formers or metabolic inhibitors in bacteria?. Nat. Rev. Microbiol..

[8] Sumi C.D., Yang B.W., Yeo I.C., Hahm Y.T. (2015). Antimicrobial peptides of the genus *Bacillus*: A new era for antibiotics. Can. J. Microbiol..

[9] Sarkar N., Paulus H. (1972). Function of peptide antibiotics in sporulation. Nat. New Biol..

[10] Frangou-Lazaridis M., Seddon B. (1985). Effect of gramicidin s on the transcription system of the producer *Bacillus brevis* Nagano. Micro..

[11] Ristow H., Pschorn W., Hansen J., Winkel U. (1979). Induction of sporulation in *Bacillus brevis* by peptide antibiotics. Nature.

[12] Özcengiz G., Öğülür I. (2015). Biochemistry, genetics and regulation of bacilysin biosynthesis and its significance more than an antibiotic. New Biotechnol..

[13] Hilton M.D., Alaeddinoglu N.G., Demain A.L. (1988). *Bacillus subtilis* mutant deficient in the ability to produce the dipeptide antibiotic bacilysin: Isolation and mapping of the mutation. J. Bacteriol..

[14] Özcengiz G., Alaeddinoglu N.G. (1991). Bacilysin production by *Bacillus subtilis*: Effects of bacilysin, pH and temperature. Folia Microbiol..

[15] Özcengiz G., Alaeddinoglu N.G. (1991). Bacilysin production and sporulation in *Bacillus subtilis*. Curr. Microbiol..

[16] Kenig M., Abraham E.P. (1976). Antimicrobial activities and antagonists of bacilysin and anticapsin. J. Gen. Microbiol..

[17] Wang T., Liu X.H., Wu M.B., Ge S. (2018). Molecular insights into the antifungal mechanism of bacilysin. J. Mol. Model..

[18] Foster J.W., Woodruff H.B. (1946). Bacillin, a New Antibiotic Substance from a Soil Isolate of *Bacillus subtilis*. J. bacteriol..

[19] Newton G.G. (1949). Antibiotics from a Strain of *Bacillus subtilis*: Bacilipin A and B and Bacilysin. Br. J. Exp. Pathol..

[20] Rogers H., Newton G., Abraham E. (1965). Production and purification of bacilysin. Biochem. J..

[21] Walker J.E., Abraham E.P. (1970). The structure of bacilysin and other products of *Bacillus subtilis*. Biochem. J..

[22] Kamiński K., Sokolowska T. (1973). The probable identity of bagilysin and tetaine. J. Antibiot..

[23] Atsumi K., Ōiwa R., Ōmura S. (1975). Production of bacillin by *Bacillus* sp. strain no. KM-208 and its identity with tetaine (bacilysin). J. Antibiot..

[24] Parker J.B., Walsh C.T. (2013). Action and Timing of BacC and BacD in the Late Stages of Biosynthesis of the Dipeptide Antibiotic Bacilysin. Biochemistry.

[25] Wu L., Wu H., Chen L., Lin L., Borriss R., Gao X. (2015). Bacilysin overproduction in *Bacillus amyloliquefaciens* FZB42 markerless derivative strains FZBREP and FZBSPA enhances antibacterial activity. Appl. Microbiol. Biotechnol..

[26] Roscoe J., Abraham E.P. (1966). Experiments relating to the biosynthesis of bacilysin. Biochem. J..

[27] Walker J.E. (1971). Antibiotic production and sporulation in *Bacillus subtilis*. Biochem. J..

[28] Khan M.A., Göpel Y., Milewski S., Görke B. (2016). Two small RNAs conserved in enterobacteriaceae provide intrinsic resistance to antibiotics targeting the cell wall biosynthesis enzyme glucosamine-6-phosphate synthase. Front. Microbiol..

[29] Kenig M., Vandamme E., Abraham E.P. (1976). The mode of action of bacilysin and anticapsin and biochemical properties of bacilysin resistant mutants. J. Gen. Microbiol..

[30] Wojciechowski M., Milewski S., Mazerski J., Borowski E. (2005). Glucosamine-6-phosphate synthase, a novel target for antifungal agents. Molecular modelling studies in drug design. Acta Biochim. Pol..

[31] Wu L., Wu H., Chen L., Xie S., Zang H., Borriss R., Gao X. (2014). Bacilysin from *Bacillus amyloliquefaciens* FZB42 has specific bactericidal activity against harmful algal bloom species. Appl. Environ. Microbiol..

[32] Perry D. (1981). Peptide transport in *Staphylococcus aureus*. J. Gen. Microbiol..

[33] Perry D., Abraham E.P. (1979). Transport and metabolism of bacilysin and other peptides by suspensions of *Staphylococcus aureus*. J. Gen. Microbiol..

[34] Steinborn G., Hajirezaei M.R., Hofemeister J. (2005). bac genes for recombinant bacilysin and anticapsin production in *Bacillus* host strains. Arch. Microbiol..

[35] Yazgan A., Özcengiz G., Marahiel M.A. (2001). Tn10 insertional mutations of *Bacillus subtilis* that block the biosynthesis of bacilysin. Biochim. Biophys. Acta.

[36] Inaoka T., Takahashi K., Ohnishi-Kameyama M., Yoshida M., Ochi K. (2003). Guanine nucleotides guanosine 5′-diphosphate 3′-diphosphate and GTP co-operatively regulate the production of an antibiotic bacilysin in *Bacillus subtilis*. J. Biol. Chem..

[37] Karata A.Y., Çetin S., Özcengiz G. (2003). The effects of insertional mutations in *com*Q, *com*P, *srf*A, *spo*0H, *spo*0A and *abr*B genes on bacilysin biosynthesis in *Bacillus subtilis*. Biochim. Biophys. Acta.

[38] Rabbee M.F., Ali M., Choi J., Hwang B., Jeong S., Baek K. (2019). *Bacillus velezensis*: A Valuable Member of Bioactive Molecules within Plant Microbiomes. Molecules.

[39] Chen X.H., Scholz R., Borriss M., Junge H., Mögel G., Kunz S., Borriss R. (2009). Difficidin and bacilysin produced by plant-associated *Bacillus amyloliquefaciens* are efficient in controlling fire blight disease. J. Biotechnol..

[40] Rabbee M.F., Baek K.H. (2020). Antimicrobial Activities of Lipopeptides and Polyketides of *Bacillus velezensis* for Agricultural Applications. Molecules.

[41] Han X., Shen D., Xiong Q., Bao B., Zhang W., Dai T., Zhao Y., Borriss R., Fan B. (2021). The plant-beneficial rhizobacterium *Bacillus velezensis* FZB42 controls the soybean pathogen phytophthora sojae due to bacilysin production. Appl. Environ. Microbiol..

[42] Wu L., Wu H., Chen L., Yu X., Borriss R., Gao X. (2015). Difficidin and bacilysin from *Bacillus amyloliquefaciens* FZB42 have antibacterial activity against *Xanthomonas oryzae* rice pathogens. Sci. Rep..

[43] Ba§alp A., Özcengiz G., Alaeddinoĝlu N.G. (1992). Changes in patterns of alkaline serine protease and bacilysin formation caused by common effectors of sporulation in *Bacillus subtilis* 168. Curr. Microbiol..

[44] Nannan C., Vu H.Q., Gillis A., Caulier S., Nguyen T.T.T., Mahillon J. (2021). Bacilysin within the *Bacillus subtilis* group: Gene prevalence versus antagonistic activity against Gram-negative foodborne pathogens. J. Biotechnol..

[45] Zaid D.S., Cai S., Hu C., Li Z., Li Y., Gralnick J.A. (2022). Comparative Genome Analysis Reveals Phylogenetic Identity of *Bacillus velezensis* HNA3 and Genomic Insights into Its Plant Growth Promotion and Biocontrol Effects. Microbiol. Spectr..

[46] Han L.-L., Liu Y.-C., Miao C.-C., Feng H. (2019). Disruption of the pleiotropic gene *sco*C causes transcriptomic and phenotypical changes in *Bacillus pumilus* BA06. BMC Genom..

[47] Caldwell R., Sapolsky R., Weyler W., Maile R.R., Causey S.C., Ferrari E. (2001). Correlation between *Bacillus subtilis sco*C phenotype and gene expression determined using microarrays for transcriptome analysis. J. Bacteriol..

[48] Barbieri G., Albertini A.M., Ferrari E., Sonenshein A.L., Belitsky B.R. (2016). Interplay of CodY and ScoC in the regulation of major extracellular protease genes of *Bacillus subtilis*. J. Bacteriol..

[49] Shank E.A., Kolter R. (2011). Extracellular signaling and multicellularity in *Bacillus subtilis*. Curr. Opin. Microbiol..

[50] Mahlstedt S., Fielding E.N., Moore B.S., Walsh C.T. (2010). Prephenate Decarboxylases: A New Prephenate-Utilizing Enzyme Family That Performs Nonaromatizing Decarboxylation en Route to Diverse Secondary Metabolites. Biochemistry.

[51] Parker J.B., Walsh C.T. (2012). Olefin Isomerization Regiochemistries during Tandem Action of BacA and BacB on Prephenate in Bacilysin Biosynthesis. Biochemistry.

[52] Mahlstedt S.A., Walsh C.T. (2010). Investigation of anticapsin biosynthesis reveals a four-enzyme pathway to tetrahydrotyrosine in *Bacillus subtilis*. Biochemistry.

[53] Parker J.B., Walsh C.T. (2012). Stereochemical Outcome at Four Stereogenic Centers during Conversion of Prephenate to Tetrahydrotyrosine by *bac*ABGF in the Bacilysin Pathway. Biochemistry.

[54] Rajavel M., Mitra A., Gopal B. (2009). Role of Bacillus subtilis *bac*B in the synthesis of bacilysin. J. Biol. Chem..

[55] Grossman A.D. (1995). Genetic Networks Controlling the Initiation of Sporulation and the Development of Genetic Competence in *Bacillus subtilis*. Annu. Rev. Genet..

[56] Comella N., Grossman A.D. (2005). Conservation of genes and processes controlled by the quorum response in bacteria: Characterization of genes controlled by the quorum-sensing transcription factor ComA in *Bacillus subtilis*. Mol. Microbiol..

[57] Deshmukh A., Gopal B. (2020). Structural insights into the catalytic mechanism of *Bacillus subtilis bac*F. Acta Crystallogr..

[58] Perego M., Glaser P., Hoch J.A. (1996). Aspartyl-phosphate phosphatases deactivate the response regulator components of the sporulation signal transduction system in *Bacillus subtilis*. Mol. Microbiol..

[59] Weinrauch Y., Penchev R., Dubnau E., Smith E., Dubnau D. (1990). A *Bacillus subtilis* regulatory gene product for genetic competence and sporulation resembles sensor protein members of the bacterial two-component signal-transduction systems. Genes Dev..

[60] Solomon J.M., Magnuson R., Srivastava A., Grossman A.D. (1995). Convergent sensing pathways mediate response to two extracellular competence factors in *Bacillus subtilis*. Genes Dev..

[61] Magnuson R., Solomon J., Grossman A.D. (1994). Biochemical and genetic characterization of a competence pheromone from *Bacillus subtilis*. Cell.

[62] Core L., Perego M. (2003). TPR-mediated interaction of RapC with ComA inhibits response regulator-DNA binding for competence development in *Bacillus subtilis*. Mol. Microbiol..

[63] Lazazzera B.A., Solomon J.M., Grossman A.D. (1997). An exported peptide functions intracellularly to contribute to cell density signaling in *Bacillus subtilis*. Cell.

[64] Lazazzera B.A., Kurtser I.G., Mcquade R.S., Grossman A.D. (1999). An autoregulatory circuit affecting peptide signaling in *Bacillus subtilis*. J. Bacteriol..

[65] Wolf D., Rippa V., Mobarec J.C., Sauer P., Adlung L., Kolb P., Bischofs I.B. (2016). The quorum-sensing regulator ComA from *Bacillus subtilis* activates transcription using topologically distinct DNA motifs. Nucleic Acids Res..

[66] Horinouchi S., Ueda K., Nakayama J., Ikeda T. (2010). Cell-to-cell communications among microorganisms. Chem. Biol..

[67] Bongiorni C., Ishikawa S., Stephenson S., Ogasawara N., Perego M. (2005). Synergistic regulation of competence development in *Bacillus subtilis* by two Rap-Phr systems. J. Bacteriol..

[68] Auchtung J.M., Lee C.A., Grossman A.D. (2006). Modulation of the ComA-dependent quorum response in *Bacillus subtilis* by multiple Rap proteins and Phr peptides. J. Bacteriol..

[69] Smits W.K., Bongiorni C., Veening J.-W., Hamoen L.W., Kuipers O.P., Perego M. (2007). Temporal separation of distinct differentiation pathways by a dual specificity Rap-Phr system in *Bacillus subtilis*. Mol. Microbiol..

[70] Köroğlu T.E., Öğülür İ., Mutlu S., Yazgan-Karataş A., Özcengiz G. (2011). Global Regulatory Systems Operating in Bacilysin Biosynthesis in *Bacillus subtilis*. J. Mol. Microbiol. Biotechnol..

[71] Pottathil M., Jung A., Lazazzera B.A. (2008). CSF, a species-specific extracellular signaling peptide for communication among strains of *Bacillus subtilis* and *Bacillus mojavensis*. J. Bacteriol..

[72] Fernandes P.A.V., Arruda I.R.D., Santos A.F.A.B.D., Araújo A.A.D., Maior A.M.S., Ximenes E.A. (2007). Antimicrobial activity of surfactants produced by *Bacillus subtilis* R14 against multidrug-resistant bacteria. Braz. J. Microbiol..

[73] Sarwar A., Hassan M.N., Imran M., Iqbal M., Majeed S., Brader G., Sessitsch A., Hafeez F.Y. (2018). Biocontrol activity of surfactin A purified from *Bacillus* NH-100 and NH-217 against rice bakanae disease. Microbiol. Res..

[74] Sabaté D.C., Audisio M.C. (2013). Inhibitory activity of surfactin, produced by different *Bacillus subtilis* subsp. *subtilis* strains, against *Listeria monocytogenes* sensitive and bacteriocin-resistant strains. Microbiol. Res..

[75] Robertson J.B., Gocht M., Marahiel M.A., Zuber P. (1989). AbrB, a regulator of gene expression in *Bacillus*, interacts with the transcription initiation regions of a sporulation gene and an antibiotic biosynthesis gene. Proc. Natl. Acad. Sci. USA.

[76] Barbieri G., Voigt B., Albrecht D., Hecker M., Albertini A.M., Sonenshein A.L., Ferrari E., Belitsky B.R. (2015). CodY regulates expression of the *Bacillus subtilis* extracellular proteases Vpr and Mpr. J. Bacteriol..

[77] Sonenshein A.L. (2005). CodY, a global regulator of stationary phase and virulence in Gram-positive bacteria. Curr. Opin. Microbiol..

[78] Kallios P.T., Fagelson J.E., Hoch J.A., Straucht M.A. (1991). The Transition State Regulator Hpr of *Bacillus subtilis* Is a DNA-binding Protein. J. Biol. Chem..

[79] Inaoka T., Wang G., Ochi K. (2009). ScoC regulates bacilysin production at the transcription level in *Bacillus subtilis*. J. Bacteriol..

[80] Kobayashi K. (2007). Gradual activation of the response regulator DegU controls serial expression of genes for flagellum formation and biofilm formation in *Bacillus subtilis*. Mol. Microbiol..

[81] Dahl M.K., Msadek T., Kunst F., Rapoport G. (1991). Mutational analysis of the *Bacillus subtilis* DegU regulator and its phosphorylation by the DegS protein kinase. J. Bacteriol..

[82] Mariappan A., Makarewicz O., Chen X.-H., Borriss R. (2012). Two-Component Response Regulator DegU Controls the Expression of Bacilysin in Plant-Growth-Promoting Bacterium *Bacillus amyloliquefaciens* FZB42. J. Mol. Microbiol..

[83] Köroǧlu T.E., Kurt-Gür G., Ünlü E.C., Yazgan-Karataş A. (2008). The novel gene *yvf*I in *Bacillus subtilis* is essential for bacilysin biosynthesis. Antonie Van Leeuwenhoek..

[84] Jiang J., Gao L., Bie X., Lu Z., Liu H., Zhang C., Lu F., Zhao H. (2016). Identification of novel surfactin derivatives from NRPS modification of *Bacillus subtilis* and its antifungal activity against *Fusarium moniliforme*. BMC Microbiol..

